# Editorial: Insights in neuroenergetics and brain health: 2024–2025

**DOI:** 10.3389/fnins.2025.1745196

**Published:** 2025-12-01

**Authors:** Avital Schurr, David Mokler, Hyoung-gon Lee, Carla Ines Tasca

**Affiliations:** 1 Department of Anesthesiology and Perioperative Medicine, School of Medicine, University of Louisville, Louisville, KY, United States; 2 Department of Pharmacology, University of New England, Biddeford, ME, United States; 3 Department of Neuroscience, Developmental and Regenerative Biology, The University of Texas at San Antonio, San Antonio, TX, United States; 4 Departamento de Bioquímica, Centro de Ciências Biológicas, Universidade Federal de Santa Catarina, Florianópolis, SC, Brazil

**Keywords:** autophagosomes, bipolar disease, disulupidptosis, glioma, lactate, micronutraceuticals, migraine, neuroenergetics

This Research Topic includes three reviews, a brief research report, and hypothesis paper. The first review is by Mason and is titled “*Say hello to my little friend… micronutraceuticals in neuroenergetics, neuronal health, and neurodegenerative diseases*.” It begins by reviewing the direct and indirect effects of vitamins and minerals (micronutraceuticals) on brain energy metabolism and the health of neurons. It then summarizes the studies that exhibit the impact of micronutraceuticals on neurodegenerative diseases, from their protective influences to the negative impact of their deficiencies. The review ends by outlining key research gaps and future directions. The second review article is by Huang et al., titled “*A Narrative Review of Autophagy in Migraine*.” The authors attempted to evaluate the possible involvement of autophagy in the pathophysiology of migraine with the aim of clarifying its role and implications for future research and therapeutic strategies. To achieve this aim, they conducted a PubMed search concluded at the end of 2024, using several key terms in different combinations. Where migraine is concerned, their search indicated that the activation of the phosphoinositide 3-kinase (PI3K)/protein kinase B (PKB/Akt) signaling pathway exerts direct influences on the mammalian target of rapamycin (mTOR) and reduces autophagy levels. The search also showed that stimulation of purinergic ligand-gated ion channel type 7 receptor (P2X7R) in microglia can hinder autophagy through interference in the fusion of autophagosomes and lysosomes, and thus impeding the degradation of substrates within the autophagolysosome. These findings suggest that autophagy may play a significant role in the pathophysiology of migraine, particularly in its development and central sensitization. The third review is by Chang et al., titled “*Disulfidptosis: a new target for central nervous system disease therapy*.” Disulfidptosis is a pathological process identified by cancer researchers, which happens with NADPH deficiency and excess disulfide bonds in cells that have high SLC7A11 expression. Glucose deprivation causes this condition, leading to disulfide stress recognized as a specific type of oxidative stress. Proteins and metabolic pathways involved in disulfidptosis are also associated with neurodegenerative diseases, neurogliomas and cerebral ischemia, though a specific mechanism responsible for this correlation remains unknown. This review demonstrates a role for Thio metabolism disruption and disulfide stress in CNS diseases. The authors highlight potential therapeutic strategies and offer a testable hypothesis that might be a promising target for treating CNS diseases. The research article by Bertocci and Diler, titled “*Lactate flux dysfunction in patients with bipolar disorder: preliminary insights from ultra-high field 7T MRSI during task and rest*,” measured the difference in lactate influx changes between a sample of 17 participants with pediatric-onset bipolar disorder (BD) and 8 healthy participants. Their results suggest that lactate flux is dysfunctional in well-characterized BD, highlighting the importance of lactate as a mechanistic marker of BD. The last entry of this Research Topic is by Schurr, titled “*Glioma neuron symbiosis: a hypothesis*,” postulates that glioma-produced lactate is the preferred oxidative energy substrate of their surrounding neurons. As a result, by using lactate, neurons bypass glycolysis, sparing their glucose and making it readily available for the glucose-craving cancer cells. Such symbiotic exchange, especially at the initial stages of malignancy, assures the budding cancer cells an ample glucose supply ahead of the development of additional vasculature. Together, the contributions in this special section offer a multifaceted exploration of neuroenergetics and brain health, highlighting emerging mechanisms, therapeutic targets, and conceptual frameworks that are shaping the field in 2024–2025. From micronutraceuticals and autophagy to disulfidptosis and lactate metabolism, collectively, these papers deepen our understanding of how energy metabolism intersects with brain health and disease. They point toward innovative strategies for diagnosis, prevention, and treatment across a spectrum of neurological and psychiatric conditions. We hope this Research Topic stimulates further research and dialogue in the dynamic and evolving field of neuroenergetics.

